# The Role of Exosomes in Pancreatic Cancer From Bench to Clinical Application: An Updated Review

**DOI:** 10.3389/fonc.2021.644358

**Published:** 2021-02-26

**Authors:** Kai Chen, Qi Wang, Marko Kornmann, Xiaodong Tian, Yinmo Yang

**Affiliations:** ^1^ Department of General Surgery, Peking University First Hospital, Beijing, China; ^2^ Clinic of General, Visceral and Transplantation Surgery, University of Ulm, Ulm, Germany

**Keywords:** pancreatic cancer, exosome, cell-to-cell communication, biomarker, therapeutic vehicle

## Abstract

Pancreatic ductal adenocarcinoma (PDAC) remains one of the most dismal gastrointestinal malignancies with an overall 5-year survival rate of 8%–9%. The intra-tumor heterogeneity and special tumor microenvironment in PDAC make it challenging to develop effective treatment strategies. Exosomes are extracellular vesicles that originate from the endosomes and have a diameter of 40–160 nm. A growing body of evidence has shown that exosomes play vital roles in tumor initiation and development. Recently, extensive application of exosomes as biomarkers and drug carriers has rendered them attractive in the field of PDAC. This review summarizes the latest progress in the methodologies for isolation, modification, and tracking of exosomes, exosome-mediated cell-to-cell communication, clinical applications of exosome as minimally invasive liquid biopsy and drugs carriers, as well as their involvement in the angiogenic regulation in PDAC. In spite of these advancements, some obstacles are still required to be overcome to use the exosome-based technologies for early diagnosis or improvement of prognosis of patients with PDAC.

## Introduction

Pancreatic ductal adenocarcinoma (PDAC) remains one of the most dismal gastrointestinal malignancies with an overall 5-year survival rate of 8%–9%, which brings great challenges for developing effective therapeutic strategies (1). Although radical excision is the only potentially curative therapy for PDAC, only 15%–20% of PDAC patients are eligible for radical excision at the time of diagnosis due to either major vascular invasion or distant metastasis (2, 3). Even after curative resection, the majority of patients still encounter local recurrence or systematic metastasis within just 12 months, with a 5-year survival rate after surgery of 20%–30% (4). Nowadays, the paradigm shift from the traditional “surgery first” approach to the modern “multi-disciplinary team (MDT)” treatment significantly improved the short-term prognosis of patients with PDAC; however this MDT approach is not sufficient enough to markedly increase long-term survival of the majority of patients with PDAC (5). Thus, it is imperative to develop new diagnostic and treatment strategies for PDAC.

**Figure 1:**
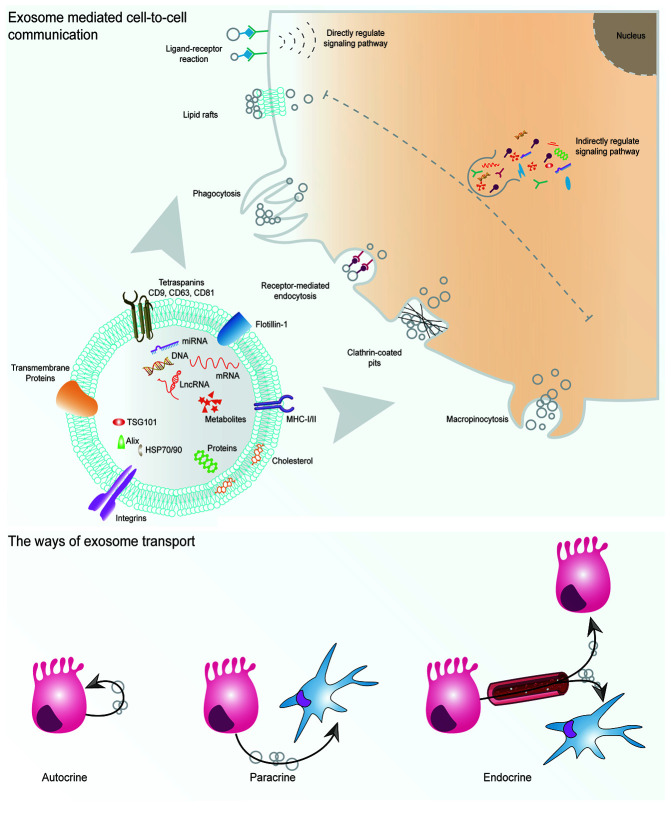
Exosomes involve intercellular communication. Exosomes derived from the parental cells affect the biological function of the recipient cells through ligand-receptor interactions and uptake of exosomal cargo. Exosome transport ways can be autocrine, paracrine, or endocrine.

Exosomes are members of the extracellular vesicle (EV) family and have an endosomal origin. Exosomes have a diameter of 40–160 nm (average, 100 nm). Under physiological or pathological conditions, all the cells inside the human body secrete exosomes into the body fluid – plasma, urine, saliva, ascites, and bile (6, 7). Similar to their parental cells, exosomes contain cell-derived biological molecules such as DNA, miRNA, mRNA, lncRNA, proteins, lipids, and metabolites (**Figure 1**). The constituents of exosomes vary a lot under different circumstances due to diverse original cell types and status. Because of the features of wide distribution and cell specificity, identification of cancer-specific exosomes *via* minimally invasive liquid biopsy might be critical for the early diagnosis, prognosis prediction and development of therapeutic strategies related to malignancies (7). Meanwhile, a growing body of evidence has revealed that the cell-to-cell communication *via* exosomes in different types of cells plays a vital role in the physiological and pathological processes such as immune response, tissue fibrosis, reproduction, tumorigenesis, and metastasis (8–11). Recently, exosomes have become a popular research area of PDAC. Studies have highlighted the clinical value of exosomes as biomarkers and drug carriers in PDAC patients (12–14). In this review, we describe the recent progress in the basic research of exosomes and discuss its clinical applications in PDAC.

## New Methodologies in Exosome Research

### Isolation of High-Quality Exosomes

**Figure 2:**
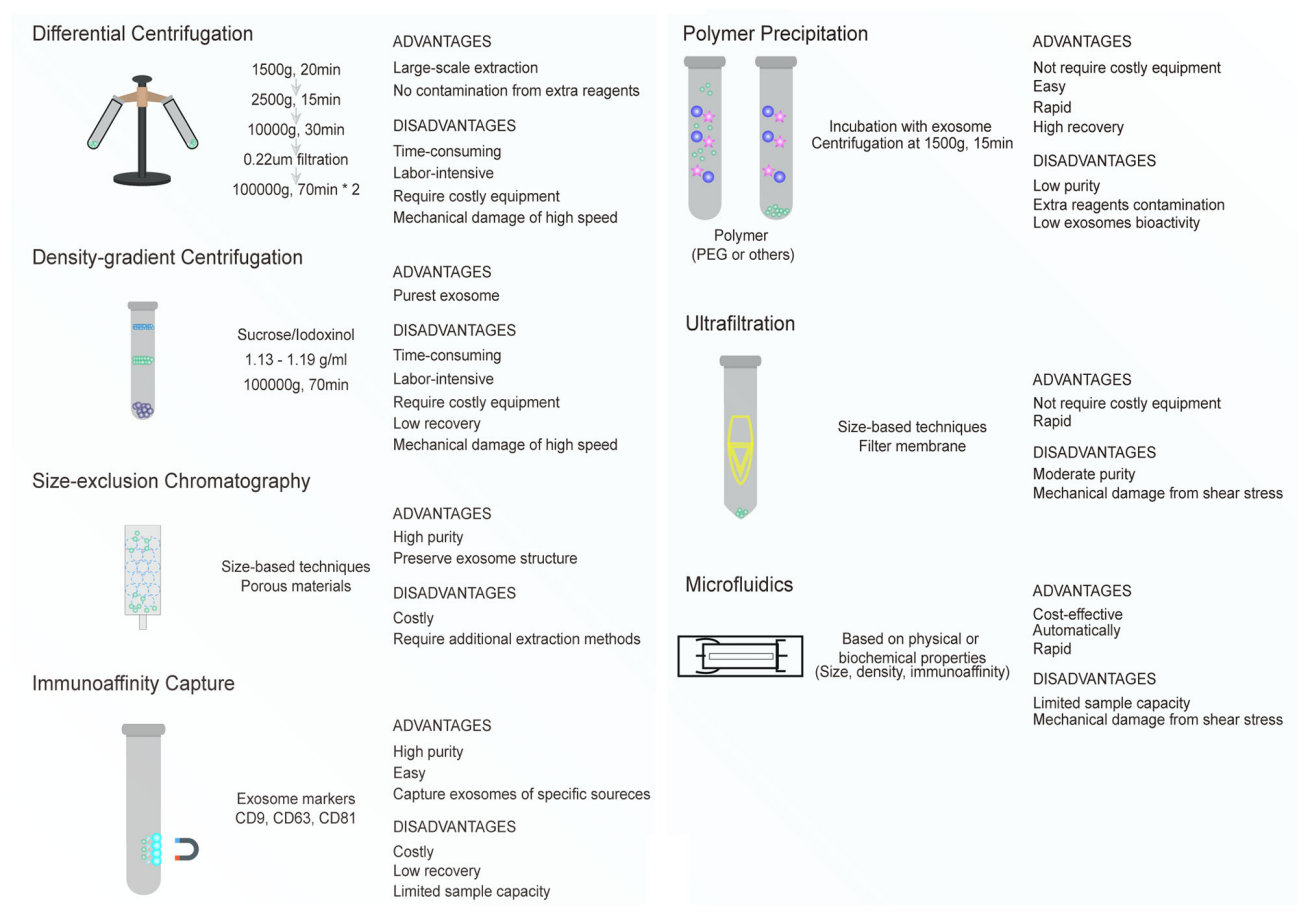
Different isolation methods of exosomes. Each method has their own advantages and disadvantages based on recovery, purity, operation, and cost.

Isolation of high-quality exosomes is a prerequisite of all the exosome-related studies. The International Society for Extracellular Vesicles (ISEV) classified the exosomes isolation strategies into four categories according to the recovery rate and specificity. Further, the society declared there is no gold standard for the isolation of exosomes at present (15). Although ISEV did not provide clear guidelines on the use of specific exosome isolation methods, it suggested that the investigators should give the detailed protocol in the ensuing scientific publications to guarantee the reliability and reproducibility of results. The most popular technology for purifying exosomes is ultracentrifugation (UC). UC is extensively used in almost all exosome-related studies. For isolating the high-quality exosomes from complicated body fluids such as plasma or urine, researchers also prefer a combination of isolation methods, including UC or polymer precipitation plus size exclusion chromatography (SEC). Unfortunately, the collection of pure exosomes is impossible because of the unavoidable contamination with soluble proteins and larger vesicles. Each isolation method has its specific advantages and disadvantages and results in heterogeneity in terms of size, surface markers, and contaminants in the isolated exosomes (**Figure 2**) (16, 17). Besides the traditional isolation methods such as UC, SEC, polymer precipitation, and immunocapture, some novel isolation technologies also yield high-quality exosomes (18, 19). Zhang and colleagues (20) developed the Asymmetric Flow Field-Flow Fractionation (AF4) technique, which can identify three diverse exosome subsets, including large exosome vesicles (Exo-L, 90–120 nm), small exosome vesicles (Exo-S, 60–80 nm), and non-membranous nanoparticles (exomeres, 35 nm). The proteomic profiling revealed that the biological function carried out by each subset of exosomes varies considerably. Thus, the AF4 can separate the specific subsets of vesicles for understanding the heterogeneity of exosomal populations. Niu et al. (21) introduced a new exosome isolation platform involving integrated microfluidic chip with a combination of the traditional immunomagnetic bead-based technology and the latest microfluidic method. This platform is automatic and more efficient, which is helpful in obtaining highly pure and intact exosomes. Moreover, this platform can also isolate a certain subset of exosomes with a specific protein marker (CD63). In addition, Lee and colleagues (22) developed an acoustic nano filter system that can separate nanoscale vesicles (<200 nm) in a continuous and contact-free way. The differential acoustic force was created on the basis of the size and density of the nanoparticles by ultrasound standing waves. This system can isolate exosomes with high separation yield and resolution. In recent years, novel purification methods to achieve high-quality isolation of exosomes have progressed rapidly. With the innovation in technology, efficient isolation of exosomes with high purity and quality should be the fundamental benchmark for exosome-related research.

### Engineering Exosomes

Exosomes modification through genetic or nongenetic methods can change exosomal components and improve the targeting capability of therapeutic agents. The techniques of engineering exosomes include modifications of nucleic acid, protein, and glycoprotein, which provide a new targeted strategy for tumor precision medicine. Because genetic manipulation is relatively easier to implement at a cellular level, the complicated exosome engineering mainly focusses on the genetic modification of the parental cells at present.

The small RNAs (siRNAs or miRNAs) can be directly inserted into the exosomes through temporary permeation of the exosomal membrane using either physical or chemical methods, thereby modifying the exosomal nucleic acids. The most common methodology of delivering the target siRNAs or miRNAs into exosomes is electroporation (23, 24). The nucleic acid-modified exosomes can serve as drug carriers because they interfere with the expression of target genes *in vitro* or *in vivo*. However, the current technique of exosomal nucleic acid modification still needs further optimization, since it often causes RNA aggregation, which limits the transfection efficiency. Meanwhile, the surface proteins of the parental cells can also be modified using gene editing methods, and the exosomes would then express these modified membrane proteins. These exosomes can be used to target specific cells or tissues and exert the required therapeutic effects with reduced off-target effects. Alvarez-Erviti et al. (23) engineered dendritic cells to express Lamp2b, an exosomal membrane protein that was fused with the neuron-specific RVG peptide to produce brain-specific exosomes. They loaded the purified brain-specific exosomes with exogenous siRNAs against BACE1 using electroporation, and successfully knocked down the specific gene in the brain of a murine Alzheimer’s disease model. Except for the membrane proteins, the ubiquitinated proteins can be sorted into the endosomal sorting complexes required for transport, and entrapped in the exosomes. Based on this sorting mechanism, Sterzenbach and colleagues (25) designed a fusion protein of Cre recombinase and WW tag that recognized the L-domain containing protein Ndfip1 and resulted in ubiquitination of the target protein (Cre). The fusion protein was successfully loaded into the exosomes, demonstrating a potential strategy to load specific proteins into exosomes.

Besides the modification of nucleic acids and proteins, changing the structure of glycoproteins present on the surface of exosomes may also significantly influence the exosomal physicochemical properties and biological functions. Royo et al. (26) reported that a modification of glycosylate complexes through the degradation of terminal sialic acid residues present on the surface of mouse liver-derived EVs resulted in the accumulation of EVs in the lungs. Moreover, Lee et al. (27) developed a targeting strategy to engineer EVs. The investigators observed that, when 3-(diethylamino) propylamine (DEAP) was anchored to EVs, the structure of EVs was maintained in physiological conditions (pH = 7.4); however, this structure collapsed in the acidic environment (pH < 7.0), and the contents inside the EVs were released. Using this strategy, they developed a pH-responsive drug vehicle using nano-sized vesicles. These modified vehicles remained stable in the blood circulation. The encapsulated drugs were released after the vesicles reached the acidic tumor microenvironment and were engulfed by the tumor cells. The results from the xenograft model have demonstrated that DEAP-EVs could significantly increase the concentration of doxorubicin inside the tumors and inhibit tumor growth effectively (27). These novel modification strategies may act as breakthroughs for exosome-mediated targeting of tumors.

### Exosome Tracking

Exosome tracking is a visualization technique to label exosomes with specific materials and investigates their bio-distribution at cellular or animal levels using optical, magnetic resonance, or radionuclide imaging. This technique opens up the possibility to measure the metabolic kinetics parameters of exosomes inside the body. The exosome tracking technique consists of three features – labeling, imaging, and data processing.

The labeling of exosomes is classified into two categories – indirect labeling and direct labeling. The indirect labeling method refers to the genetic manipulation, and the modification of metabolites and membranes of the parental cells. The direct labeling method includes click-chemistry-based lipophilic staining and membrane modification for purified exosomes. Investigators have constructed a fusion protein using eGFP, luciferase, and tetraspanins (CD9, CD63, and CD81– anchored on the surface of exosomes), which was then expressed inside the parental cells to mark the exosomes and enable their tracing *via* an imaging system (28–30). Recently, Tung and colleagues (31) reported a facile exosome labeling strategy. They added tetra-acetylated N-azidoacetyl-D-mannosamine (Ac4ManNAz) to the culture medium. Ac4ManNAz was spontaneously incorporated into the process of glycometabolism and loaded into the exosomes. These azido-containing exosomes were then conjugated with fluorescent dyes *via* click reaction, so the distribution of the labeled exosomes can be observed *in vivo*. Busato et al. (32) developed an innovative exosome labeling approach based on magnetic resonance imaging (MRI). The adipose stem cells (ASCs) were incubated with ultrasmall superparamagnetic iron oxide nanoparticles (USPIO, 4–6 nm) for 72 h. Then the ASCs-derived exosomes labeled with these nanoparticles were visualized *via* MRI. Further, their morphological and physiological features were also preserved. These indirect exosome labeling methods by modifying parental cells have little effect on exosomal properties. However, the efficiency is usually lower, and the procedure is more complex and time-consuming as compared with the direct labeling method using purified exosomes. The most common method of labeling purified exosomes is directly incubating the exosomes with lipophilic fluorescent dyes such as PKH67 and Dio to uniformly stain the exosomal membrane. However, most of these lipophilic dyes tend to aggregate into a mass, thus reducing the imaging quality. So, the aggregation effect must be treated carefully. Furthermore, a recent study showed that gold-carbon quantum dots (GCDs), a novel fluorescent nanomaterial, can serve as a labeling dye for tracing exosomes. GCDs could conjugate with antibodies and label the exosomes *via* the antigen-antibody reaction. Using this exosome-specific nanoprobe, investigators successfully analyzed the tracks of labeled exosomes after the exosomes were engulfed by live cells (33).

The dynamic visualization of the distribution and biological process of exosomes in high resolution *in vitro* and *in vivo* is vital. Real-time imaging for nano-sized vesicles poses a challenge for the spatial and temporal resolution of imaging systems. MRI has a great advantage in spatial resolution as compared with traditional optical imaging. In addition, the latest exosome-tracking method based on radionuclide imaging holds a great promise for dynamic detection of the bio-distribution of exosomes. Hwang et al. (34) used SPECT/CT to continuously observe the distribution of macrophage-derived exosomes labeled with (99m) Tc-HMPAO under physiological conditions. The investigators observed the redistribution of labeled exosomes from liver to brain.

Most of the current imaging techniques for exosome tracking are adapted from the mature cell tracking or medical imaging protocols and lack the specific imaging platform. The multimodal exosome imaging systems are being developed to integrate the advantages of optical, magnetic resonance, and radionuclide imaging (34). These systems can improve the quality of image reconstruction, broaden the scope of their applications, and hence, would play a significant role in the field of exosome research.

### Role of Exosomes on Tumor Microenvironment – Cell Messengers

Many studies have indicated that exosomes participate in the process of tumorigenesis and tumor progression. Nowadays, researchers are trying to explore the *in vivo* biodistribution, content heterogeneity, and biological function of these nano-sized vesicles. The exosomes originating from different cells inside a tumor have built up a unique tumor nano environment (TNE) and act as significant cell-to-cell communication mediators. The living cells shed a large number of exosomes, not only to communicate with themselves and adjacent cells through autocrine and paracrine mechanisms, but also to communicate with distant tissues or organs, playing a regulatory role, through endocrine signaling (**Figure 1**). *Via* shedding exosomes, cancer cells promote their own proliferation and migration (7). The low-grade malignant chemosensitive tumor cells may develop a malignant and chemoresistant phenotype after endocytosing exosomes from the high-grade malignant chemoresistant cells (35, 36). Moreover, tumor metastasis model experiments indicated that the exosomes from primary tumor location traveled to the target organs such as the liver and brain by the circulatory system and induced pre-metastasis niche formation, resulting in increasing the possibility of tumor metastasis (37, 38). The exosomes mediate cell-to-cell communication primarily in two ways: (A) The specific proteins on the surface of exosomes directly regulate the signaling pathway inside the recipient cell *via* receptor-ligand interaction; (B) The recipient cells engulf the exosomes loaded with miRNAs, proteins or metabolites through receptor-mediated endocytosis, clathrin-coated pits, lipid rafts, phagocytosis, or macropinocytosis, then these payloads involve intracellular signaling regulation (**Figure 1**) (39).

The physiological significance of cells shedding the exosomes remains largely unclear. Early studies hypothesized that similar to garbage bags, exosomes help in the removal of excess waste products from the cell to maintain cellular homeostasis (40). It is hard to discern if the package of exosomal constituents is accurately controlled by the specific sorting system or random assortment. However, nowadays researchers have confirmed that exosome contents play a vital role in cell-to-cell interaction, among which miRNAs are the most widely studied components (41). MiRNAs are small and endogenous non-coding RNA molecules containing about 19-24 nucleotides, which completely or partially bind the 3’ UTR within mRNA *via* base-pairing principle, resulting in target gene silencing or degradation in the post-transcriptional level (42). Recently, a growing body of studies has revealed that the cell-to-cell communication networks mediated by exosomal miRNAs act as cell messengers in PDAC, highlighting the complex tumor microenvironment of PDAC (**Table 1**). Wang et al. (43) reported that the exosomes derived from hypoxic pancreatic cancer cells (PCCs) could be engulfed by macrophages and release miR-301a to induce M2 polarization *via* activation of PTEN/PI3K signaling pathway. The macrophages with the M2 phenotype promoted malignant behaviors in pancreatic cancer cells (PCCs) by secreting TGFβ, IL10, and arginase in return. Natural killer (NK) cells can regulate the expression level of IL-26 in PCCs by shedding exosomes loaded with miR-3607-3p and inhibiting pancreatic cancer progression *in vitro* and *in vivo* (44). Exosomes loaded with miR-210 mediate the horizontal transfer of a drug-resistant phenotype from gemcitabine-resistant PCCs to chemosensitive PCCs (36). Cancer-associated fibroblast (CAF) derived exosomal miR-106b enhanced the proliferation and gemcitabine resistance of PCCs by directly targeting TP53INP1 (45). Activated pancreatic stellate cells (PSCs) continuously released exosomes containing high levels of miR-21. PCCs internalize these exosomes, resulting in the upregulation of miR-21. PSC-derived exosomal miR-21 was able to promote epithelial-to-mesenchymal transition (EMT), migration, and enhanced Ras/ERK signaling pathway activity in PCCs (46). Exosomal miR-194-5p shed from the dying tumor cells under radiotherapy was found to induce G1/S arrest and promote DNA damage repair of residual tumor repopulating cells (TRCs) to potentiate pancreatic cancer repopulation (47). In summary, exosomal miRNA mediates complicated cell-to-cell communication network inside the PDAC microenvironment involving PCCs, NK cells, macrophages, and CAFs. However, the interaction mechanisms involving other components such as endothelial cells (ECs) in PDAC are currently unknown. Further research is needed to study the bi-directional communication among these components in PDAC, which even forms a positive feedback loop for promoting the tumor progression. Besides miRNAs, LncRNAs and proteins in exosomes, although with low abundance, also play a pivotal role in PDAC microenvironment.

**Table 1:** Exosome miRNA-mediated cell-to-cell communication network in pancreatic ductal adenocarcinoma (PDAC) microenvironment.

Parental cell	Recipient cell	Cargo	Isolation method	Culture conditions	Biological function	Reference
PCCs	Macrophages	miR-301a	Ultracentrifugation	Hypoxia	Induce M2 polarization	(43)
NKs	PCCs	miR-3607	Ultracentrifugation	Normoxia	Inhibit progression	(44)
Gemcitabine-resistant PCCs	Chemosensitive PCCs	miR-210	ExoQuick-TC	Normoxia	Transfer of drug-resistant phenotype	(36)
CAFs	PCCs	miR-106b	ExoQuick-TC	Normoxia	Promote proliferation and Gemcitabine resistance	(45)
PSCs	PCCs	miR-21	Ultracentrifugation	Normoxia	Induce EMT	(46)
Dying tumor cells	TRCs	miR-194-5p	Ultracentrifugation	Normoxia	Promote G1/S arrest and DNA damage repair	(47)

PCCs, Pancreatic cancer cells; NKs, Natural killer cells; CAFs, Cancer-associated fibroblasts; PSCs, Pancreatic stellate cells; EMT, Epithelial-to-mesenchymal transition; TRCs, Tumor repopulating cells.

## Clinical Applications of Exosomes

### Exosomes as Biomarkers for Early and Non-Invasive Diagnosis of PDAC

**Figure 3:**
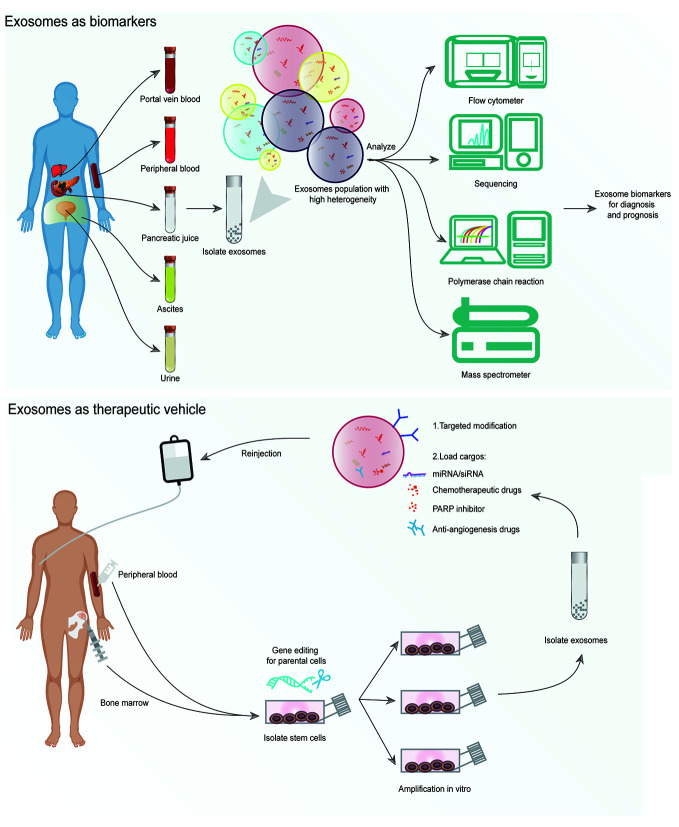
The clinical application of exosomes in pancreatic cancer. Exosomes are isolated from complex body fluids, including portal vein blood, peripheral blood, pancreatic juice, ascites, and urine. Exosomal miRNAs or proteins are identified as biomarkers for early diagnosis and the evaluation of prognosis. It is feasible to collect clinical-grade exosomes on a large scale to culture stem cells. The strategy of using exosomes as drug carriers holds significant therapeutic value when combined with exosome modification techniques.

The blur clinical signs and symptoms of PDAC result in a very low diagnosis rate during the early stages. Moreover, the current diagnostic techniques are insufficient to screen out early asymptomatic patients, and the serum tumor markers of PDAC, such as carbohydrate antigen 19-9 (CA19-9) and carcinoembryonic antigen (CEA), have limited specificity and sensitivity. Thus, the development of new and reliable biomarkers of PDAC is critical to improve the early detection and radical resection rates. Recently, the new liquid biopsy strategy mediated by exosomal markers has showed potential value as a non-invasive diagnostic method (**Figure 3**). Under the protection of endogenous membrane of the exosomes, the diagnostic markers can remain stable inside the blood circulation, which makes the diagnosis more reliable. Therefore, this strategy may become crucial for the non-invasive diagnosis of PDAC in the near future.

Exosomes loaded with multiple diagnostic molecules can be isolated from different types of body fluids, making the exosomal markers-based liquid biopsy more attractive for early tumor detection, tumor progression monitoring, and prognosis assessment. Many studies have highlighted the possibility of clinical translation of exosomal biomarkers in PDAC (**Table 2**). Plasma exosomal miR-21 could be used to differentiate patients with PDAC, intraductal papillary mucinous neoplasm (IPMN) and healthy participants (HP) (48, 49). Goto et al. (50) reported that exosomal miR-21 isolated from pancreatic juice using ultracentrifugation could also differentiate PDAC and chronic pancreatitis (CP). The patients with PDAC had a higher level of exosomal miR-451 than the HP, and the expression level of miR-451 wassignificantly correlated with recurrence and survival time (48, 51). Plasma exosomal miR-196a and miR-1246 also showed diagnostic value for localized pancreatic cancer (52). Moreover, the combination of multiple exosomal biomarkers to create a predictive model significantly improved the accuracy of diagnosis and prognosis (53, 55). In a clinical study with large cohorts, Melo et al. (54) found that Glypican-1 (GPC1), a cell surface proteoglycan, was specifically enriched in tumor cell-derived exosomes, and GPC1+ exosomes in the serum served as a non-invasive diagnostic and screening biomarker with absolute sensitivity and specificity (AUC = 1.0). GPC1+ exosomes could also distinguish patients with early and late PDAC from HP and patients with benign pancreatic disease (BPD) (56–58).

**Table 2:** Exosomes as biomarkers for diagnosis and prognosis of pancreatic ductal adenocarcinoma (PDAC).

Exosomal cargo	Body fluid	Isolation method	Sample size	Clinical application	Reference
miR-21	PVB and PB	Ultracentrifugation	55 PDAC	Evaluation of recurrence and prognosis	(48)
	PB	ExoQuick-TC	32 PDAC, 29 IPMN, and 22 HP	Early diagnosis	(49)
	Pancreatic juice	Ultracentrifugation	27 PDAC and 8 CP	Early diagnosis	(50)
miR-451a	PVB and PB	Ultracentrifugation	55 PDAC	Evaluation of recurrence and prognosis	(48)
	PB	Ultracentrifugation	56 PDAC and 3 HP	Evaluation of recurrence and prognosis	(51)
miR-196a, miR-1246	PB	ExoQuick-TC	15 PDAC and 15 HP	Screen localized PDAC	(52)
Panel: CD44v6, Tspan8, EpCAM, MET, CD104 and miR-1246, miR-4644, miR-3976, miR-4306	PB	Sucrose-gradient centrifugation	131 PDAC, 25CP, 22 BPD and 42 HP	Early diagnosis	(53)
Glypican-1	PB	Sucrose-gradient centrifugation	246 PDAC, 24 CP, 5 IPMN, 8 BPD, and 20 HP	Early screening tool and evaluation of tumor burden and prognosis	(54)

PVB, Portal vein blood; PB, Peripheral blood; PDAC, Pancreatic adenocarcinoma; IPMN, Intraductal papillary mucinous neoplasm; HP, Healthy participants; CP, Chronic pancreatitis; BPD, Benign pancreatic disease.

In summary, the exosome-mediated non-invasive diagnosis strategy may overcome the shortages of traditional serum tumor markers for early detection of PDAC. However, a single exosomal marker used for diagnosis is usually associated with high specificity and low sensitivity (59). Thus, comprehensive diagnostic strategies combining exosomal miRNAs, proteins and traditional serum tumor markers are urgently needed to improve the specificity and sensitivity of PDAC diagnosis.

### Exosomes as a Therapeutic Vehicle of PDAC

In recent years, researchers have made great progress in the development of exosomes as drug carriers (60, 61). As compared with liposomes and other nanoparticles, exosomes possess better biocompatibility as drug carriers (62). Injected exosomes shed from endogenous cells of the body are tolerated with minimal immune reaction and toxicity (63, 64). The cargos can be efficiently delivered into the tumor microenvironment using exosomes since these vesicles have the ability to penetrate the blood-tissue barrier. For instance, Alvarez-Erviti et al. (23) demonstrated that the self-derived exosomes were able to deliver siRNA to the brain through the blood-brain barrier. The therapeutic exosomes were found to be taken up by the target tissues in mice with low immune clearance rate *via* intravenous injection (55, 63). Mesenchymal cells- or epithelial cells- derived exosomes did not cause toxic side effects even after being repeatedly injected in mice (14). Kordelas et al. (65) isolated exosomes from the mesenchymal stem cells (MSCs) to treat graft-versus-host disease (GvHD) and found that the exosomes were well tolerated.

Since exosomal miRNAs have the potential capability to suppress the expression of target genes in recipient cells, investigators have tried to engineer the exosomes by loading target specific miRNA or siRNA to block the abnormal signaling pathways in PDAC cells in recent years. With the protection of the bilayer lipid membrane, exosomal RNAs can be safely transported to the lesion sites without any degradation by natural ribonucleases in the blood (66). The first clinical-grade MSCs-derived exosomes loaded with siRNA against Kras^G12D^ was reported in 2017, which served as a promising therapeutic strategy in PDAC animal models (13). By targeting Kras^G12D^ mutation of PDAC cells *in vivo*, these engineered exosomes showed a significantly increased overall survival without any toxicity. Moreover, this strategy has found its way to a Phase-I clinical trial in PDAC patients with Kras^G12D^ mutation (NCT03608631).

In order to develop exosomes with a better target ability, we can conduct the modification of exosomes by direct or indirect methods, as discussed previously. For example, mouse immature dendritic cell-derived exosomes loaded with doxorubicin showed targeted αv integrin positive cancer cells with high efficacy, though engineering exosomes to express a fusion protein of Lamp2b and αv integrin-specific RGD (67). Thus, to achieve high targeting of PDAC tumor cells, a new therapeutic strategy can be developed by engineering exosomes and loading specific payloads such as siRNAs, inhibitors, or chemotherapy drugs followed by verifying the safety and efficacy of the exosomes in organoid and patient-derived tumor xenograft models.

### Exosomes and Tumor-Associated Neovasculature in PDAC

Tumor-associated neovasculature helps tumor cells in acquiring nutrients and oxygen and clearing metabolic wastes efficiently (68, 69). In the process of tumor development, angiogenesis-related signaling pathways are highly activated to support the continued growth of tumor lesions, which pave the way for local invasion and distant metastasis of tumor cells. However, PDAC is characterized by a lower microvascular density (MVD) with a high desmoplastic stromal reaction as compared with other tumors. The desmoplastic reaction results in a high pressure and collapse of the vascular structure inside PDAC. Thus, the limited vascular bed causes severe hypoxia stress in the tumor cells (70, 71). In order to adapt to the hypoxic environment, the endothelial cells (ECs) in PDAC develop hairy-like base microvilli to extend the vascular surface area, enhancing the glucose uptake rate. In addition, the basement membranes of blood vessels usually lose their integrity and develop various abnormal features such as variable diameters, excessive branching, and destroyed inter-endothelial junctions (69). All these features increase the possibility of early tumor metastasis. Thus, anti-angiogenesis therapy may bring hope for patients with PDAC. Unfortunately, the underlying mechanism of how the PDAC cells regulate angiogenesis is still not fully understood. Some clinical trials have demonstrated that anti-angiogenesis therapies failed to improve the prognosis of patients with PDAC (72–75). The complex tumor microenvironment and cell-to-cell communication among different components may contribute to the angiogenic regulation network in PDAC. Serving as a cell messenger, exosomes may play an essential role in cell-to-cell communication between ECs and other cells.

Accumulating evidences have suggested that angiogenesis inside tumors is regulated by cell-to-cell communication between ECs and other components of the tumors, including tumor cells, CAFs, and tumor-infiltrating lymphocytes (TILs), through soluble cytokines, gap junctions, and physical contact (68). Stromal cells and TILs were found to promote tumor growth *via* secreting VEGF (76). Masamune et al. (77) found that PSCs in the hypoxic environment release multiple angiogenic factors such as VEGF, MMP9, IL-8, and FGF-2 to induce ECs proliferation, migration, and angiogenesis *in vitro* and *in vivo*. In recent years, exosome-mediated cell-to-cell communications between ECs and other components inside tumors have attracted considerable attention (78). Hsu and colleagues (79) found that lung cancer derived exosomal miR-23a under hypoxic condition could inhibit the expression of PHD and ZO-1, resulting in an increase in angiogenesis and vascular permeability. Umezu et al. (80) demonstrated that exosomal miR-135b shed from hypoxic multiple myeloma cells enhanced angiogenesis *via* targeting HIF-1α. In addition, hypoxic glioblastoma derived exosomes were found to contain multiple angiogenic factors such as VEGFA, to promote the proliferation of ECs and increase the permeability of the blood-brain barrier (81, 82). However, in the field of PDAC, exosome-mediated interactions between ECs and other cells have not been elucidated. Fully understanding of these interactions under hypoxia is critical for the investigation of the special angiogenic regulation in PDAC, which will also help develop new anti-angiogenesis therapeutic strategies.

## Conclusions

PDAC is still one of the most lethal human cancers. The development of novel biomarkers and therapeutic targets is essential to improve the prognosis of patients with PDAC. Exosomes are becoming a promising tool for the early detection, prognosis assessment, and even therapeutic modality of PDAC. The studies on exosomes have progressed very rapidly in recent years. In this review, we have summarized the latest progress in the methodologies for isolation, modification, and tracking of exosomes, exosome-mediated cell-to-cell communication, clinical applications of exosome as minimally invasive liquid biopsy and drugs carrier, and their contribution to the angiogenic regulation in PDAC. Despite a lot of advancements, enormous challenges also exist. Firstly, there is still no gold standard for the isolation and identification of exosomes. The reported methods for purifying exosomes in reported studies vary a lot, making the results less reproducible or convincing. Secondly, the development of ideal exosome isolation strategies with high purity and efficiency is currently unachievable and hence clinical-grade exosomes are difficult to acquire on a large scale. Most of the exosome engineering applications for the treatment of PDAC are only limited to cell or animal experiments. Thirdly, biogenesis and sorting mechanisms for exosomes have to be further explored to efficiently engineer exosomes with specific nucleic acids, proteins, and even exogenous drugs. Finally, most of the recent exosome-related mechanistic studies were conducted in normoxic conditions that only involved cancer cells. These situations do not represent the actual hypoxic microenvironment and the complicated components of PDAC. Considering the fact that exosome-mediated cell-to-cell communications among the different entities in PDAC may form a feedback loop instead of unidirectional signaling transmission, *in vitro* experimental results should be verified using animal models, or in patients with PDAC. In conclusion, there are still a few obstacles to be overcome before exosome-based technologies can be used for early diagnosis or improving the prognosis of patients with PDAC.

## Author Contributions

KC designed this review and drafted the manuscript. KC and QW searched the related literature. KC prepared the tables and figures. MK, XT, and YY revised and polished the manuscript, and approved to submit manuscript. All authors contributed to the article and approved the submitted version.

## Funding

This study was supported by The Natural Science Foundation of China (NO. 81672353 and 81871954) and the Interdisciplinary Clinical Research Project of Peking University First Hospital.

## Conflict of Interest

The authors declare that the research was conducted in the absence of any commercial or financial relationships that could be construed as a potential conflict of interest.
